# Uremic serum induces prothrombotic changes in venous endothelial cells and inflammatory changes in aortic endothelial cells

**DOI:** 10.1080/0886022X.2021.1890617

**Published:** 2021-03-01

**Authors:** Patrycja Sosińska-Zawierucha, Andrzej Bręborowicz

**Affiliations:** Department of Pathophysiology, Poznan University of Medical Sciences, Poznan, Poland

**Keywords:** Aortic endothelium, venous endothelium, uremia, thrombosis, inflammation

## Abstract

**Background:**

Uremia induces various pathologic changes in the endothelium. However, there is limited information about the differences of these effects in endothelial cells originating from different parts of the vascular tree.

**Methods:**

The effect of uremic serum obtained from patients with end stage renal failure on the gene expression and secretory activity of venous endothelial cells (VEC) and aortic endothelial cells (AEC) was studied in *in vitro* culture.

**Results:**

In VEC, the expression of genes regulating the synthesis of von Willebrand factor (vWF) was increased by 254% (*p*<.005), vascular endothelial growth factor (VEGF) synthesis by 150% (*p*<.001), tissue plasminogen activator (t-PA) synthesis by 62% (*p*<.005), platelet endothelial cell adhesion molecule by 89% (*p*<.005), and the expression of gene regulating interleukin-6 (IL-6) synthesis was reduced. In AEC, the expression of the gene regulating synthesis of IL-6 was increased by 174% (*p*<.001), and the expression of the other genes was reduced. The secretion of IL-6 was reduced in VEC by 38% (*p*<.01) and increased in AEC by 55% (*p*<.005). In VEC, increased synthesis of VEGF 64% (*p*<.001) vWF (+34%, *p*<.01), and t-PA (+53%, *p*<.002) was observed, and in AEC it was reduced.

**Conclusions:**

VEC and AEC respond in different ways after exposure to uremic serum. VEC acquires the prothrombotic phenotype, whereas in AEC the inflammatory phenotype appears.

## Introduction

Uremia causes dysfunction of endothelial cells, which is one of the main causes of cardiorenal syndrome in end stage renal failure patients [1]. Impaired function of endothelial cells is the result of the combined effect of various factors present in the uremic patient. The most important are hypertension, inflammation, oxidative stress, dyslipidemia, and asymmetric dimethylarginine [2]. Additionally, renal replacement therapy, due to its relatively low biocompatibility, can also contribute to endothelial dysfunction [3].

Evaluation of endothelial function *in vivo* is based on indirect methods, mainly measurement of the flow-mediated dilation of the arteries, which reflects the vasodilatory action of the endothelium [4]. Another approach is the measurement in blood concentration of biomarkers reflecting endothelial dysfunction, such as soluble vascular adhesion molecule-1, soluble E-selectin, or von Willebrand factor (vWF) [5]. Finally, the cytotoxicity of uremic serum toward endothelial cells can be studied *ex vivo* on human endothelial cells maintained in *in vitro* culture. Such experiments are performed mainly with human umbilical venous endothelial cells (VEC) [6–8]. It is well known that the morphology and functional properties of venous and arterial endothelial cells are totally different [9,10]. Aortic endothelial cells (AEC) are more active, as reflected by protein synthesis, whereas venous cells are larger and more pleomorphic [9]. Venous endothelial cells produce more prostacyclin than aortic cells, when studied *in vitro* under static conditions or in a mechanically active environment imitating shear stress [11]. One can expect that the effect of various noxious factors such as uremia on these cells may be different.

The goal of the present study is the comparison of the effect of uremic serum on functional properties of human venous and arterial endothelial cells studied in *in vitro* culture.

## Materials and methods

During the study, samples of serum collected from 11 patients with end stage renal failure and treated with hemodialysis were studied. The protocol of the study was approved by the University Ethical Committee (decision UMP 925/16). All patients were informed about the study, and oral consent was obtained from each person. Serum was collected before the start of the hemodialysis session. Mean age of the studied patients was 51.7 ± 11.1 years and duration of the hemodialysis therapy was 23.7 ± 10.3 months.

Collected serum samples were stored at –86 °C, until they were tested on human aortic endothelial cells (HAEC) and human umbilical vein endothelial cells (HUVEC). HAEC and HUVEC were purchased from Life Technologies Corporation (Carlsbad, CA). Cells were cultured *in vitro* in medium M200 supplemented with 2% fetal bovine serum (FBS), hydrocortisone 1 µg/mL, heparin 10 µg/mL, human epidermal growth factor 10 ng/mL, and basic fibroblast growth factor 3 ng/mL. Experiments were performed on cells monolayers in 24-well (for study of the cells’ secretory activity) or six-well (for study of gene expression) plates.

During experiments, cells were exposed for 24 h to the culture medium supplemented with the uremic serum (20%) or to the 20% pooled non-uremic serum. No signs of toxicity, as reflected by the cells morphology, cells number was observed after such treatment.

### Gene expression analysis

RNA was isolated from cells grown in six-well plates, after 24 h’ exposure to the studied sera. From each sample, one microgram of RNA was reversed-transcribed to cDNA using the iScript cDNA Synthesis Kit (Bio-RaD, Hercules, CA). Expression of genes in HAEC and HUVEC cells was examined using the real-time PCR method, according to the methods used in our lab [12]. Relative levels of the mRNA of five genes of interest: interleukin-6 (IL-6), vWF, vascular cell adhesion protein 1 (VCAM), vascular endothelial growth factor (VEGF), and platelet endothelial cell adhesion molecule (PECAM) were analyzed using SsoAdvanced™ Universal. SYBR^®^ Green Supermix (Bio-Rad, Hercules, CA) and normalized to the levels of internal house-keeping genes: hypoxanthine-guanine phosphoribosyltransferase 1 (HPRT1) and glyceraldehyde-3-phosphate dehydrogenase (GAPDH). After completed real-time PCR reactions, a melting curve analysis was performed to verify the specificity of the amplicons. Expression of the genes was calculated using the relative quantification method (2^–ΔΔCt^) [13].

### Study of the cells’ secretory activity

HAEC and HUVEC were cultured in 24-well culture plates. Confluent cells monolayers were incubated during 24 h in medium supplemented with sera (20%) from 11 uremic patients or with control nonuremic serum (20%). Then, supernatants were removed from all wells and during the next 24 h cells were cultured in serum free medium. Afterwards, supernatants were harvested from all wells for measurement of the studied molecules:

- Interleukin 6 (Elisa kit, Sigma-Aldrich, St. Louis, MO);
- Von Willebrand Factor (Elisa kit, Sigma-Aldrich, St. Louis, MO);
- Vascular endothelial growth factor (Elisa kit, R&D, Minneapolis, MN);
- Tissue plasminogen activator (t-PA) (Elisa kit, R&D, Minneapolis, MN).

The secretory activity of the cells was presented per amount of the cellular protein. Cells were lysed with 0.1 N NaOH, and the concentration of the total protein in the lysate was measured with the Quick-Start Bradford Protein Assay (Bio-Rad Laboratories, Hercules, CA).

### Statistical analysis

Results from the study are presented as mean + SD. The following statistical tests were used: *t*-test or ANOVA Kruskal–Wallis test. A *p* value lower than .05 was considered statistically significant.

## Results

**Figure 1.**
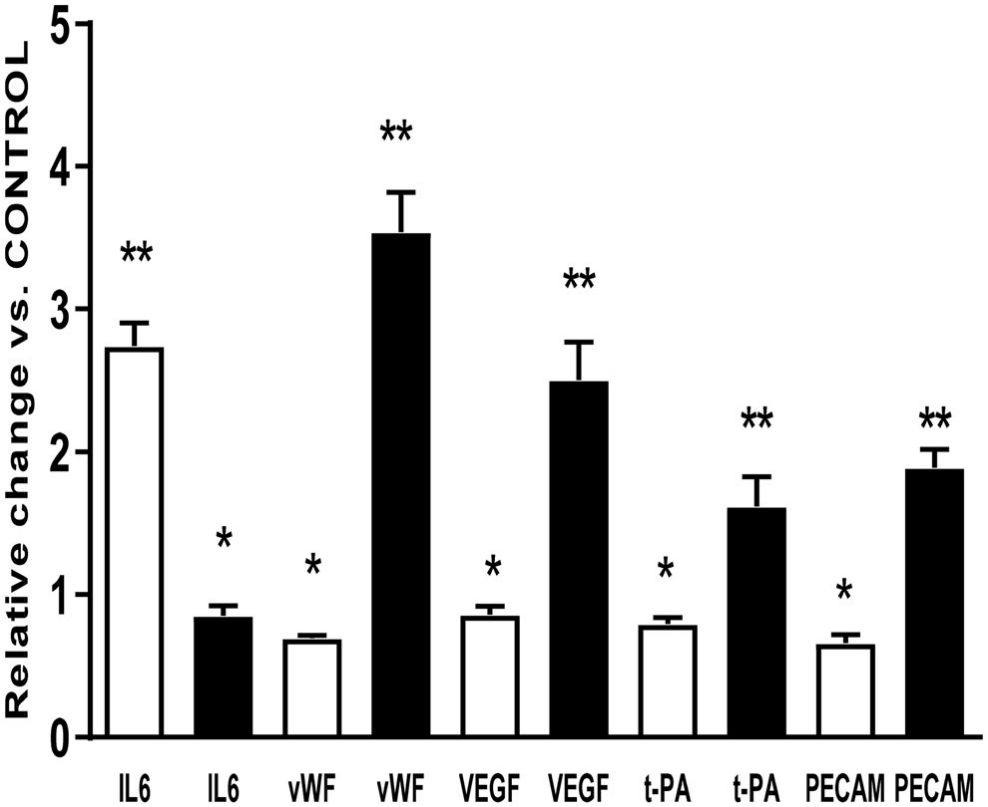
Relative expression of genes in HAEC (white bar) and HUVEC (black bar) regulating synthesis of IL6, vWF, VEGF, t-PA, and PECAM in cells exposed to 20% uremic serum. Statistical analysis was performed vs. genes expression in cells treated with 20% control serum (*p<.05; **p<.005) (*n* = 6).

Differences between the studied genes expression in HUVEC and HAEC in standard culture conditions are shown in Table 1. In HUVEC, relatively low expression of t-PA and high expression of vWF were observed, as compared to the HAEC line. Exposure of human endothelial venous and arterial cells to uremic serum resulted in changes of expression of the majority of the studied genes. However, the observed effects were not identical in both types of the cells (Figure 1). In VEC, the expression of genes regulating the synthesis of vWF was increased by 254% (*p*<.005), VEGF synthesis by 150% (*p*<.001), t-PA synthesis by 62% (*p*<.005), and PECAM by 89% (*p*<.005), and only the expression of the IL-6 gene was reduced (–15%, *p*<.05). In AEC, only the expression gene regulating the synthesis of IL-6 was increased by 174% (*p*<.001), and the expression of the other genes was reduced as compared to the control: vWF by 31% (*p*<.05), VEGF by 14% (*p*<.05), t-PA by 31% (*p*<.05), and PECAM by 34% (*p*<.05) (Figure 1).

**Table 1.** Characteristic of baseline gene expression in HUVEC and HAEC cell line.

Gene name	HUVEC vs. HAEC (fold change)	*p* Value
IL6	0.91	<.001
PECAM	1.18	<.001
t-PA	0.01	<.001
VEGF	0.17	<.001
vWF	3.05	<.001

**Figure 2.**
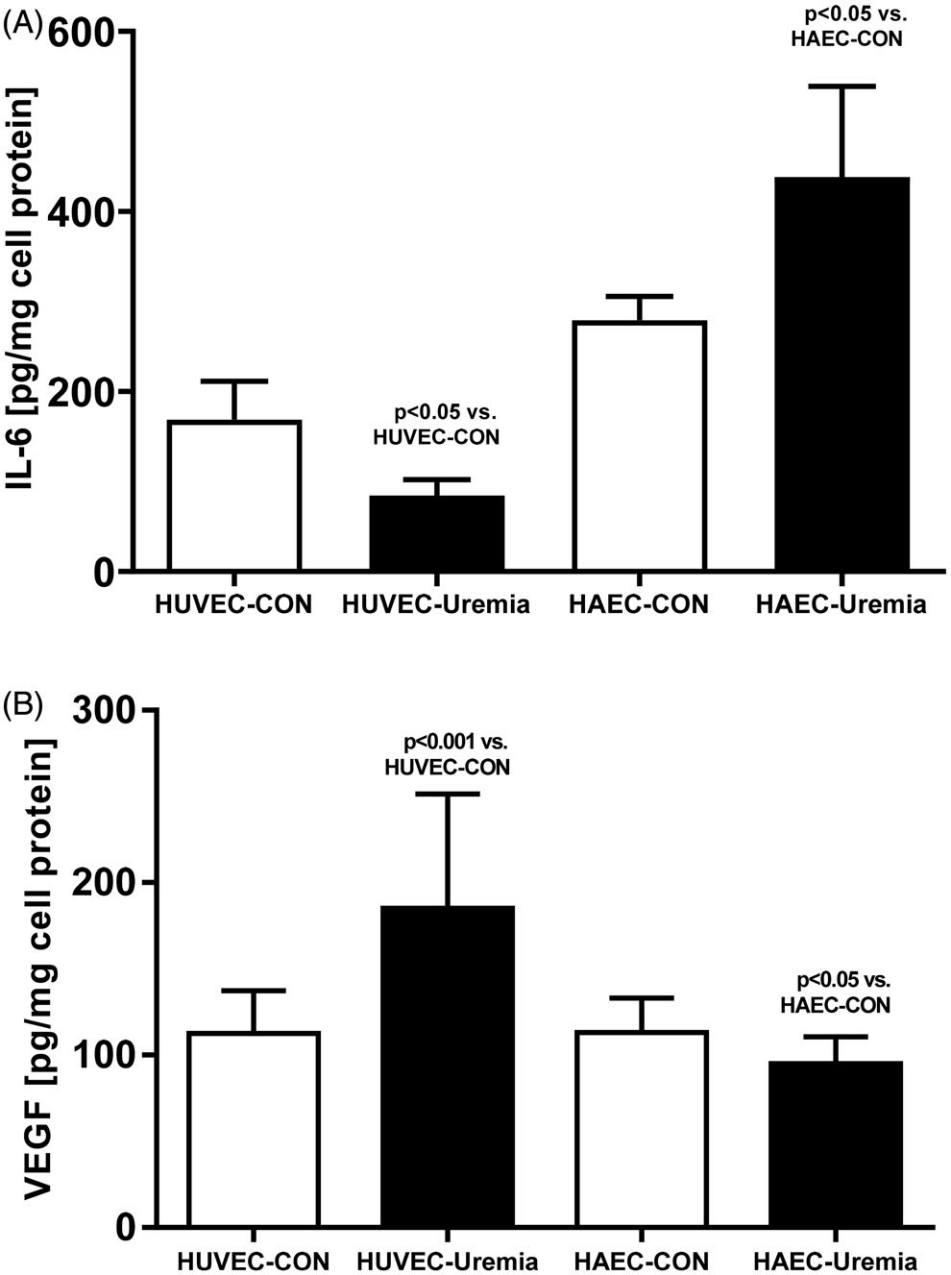
Synthesis of IL6 (A) and VEGF (B) in human umbilical endothelial cells (HUVEC) and human aortic endothelial cells (HAEC) after exposure to uremic serum (uremia) as compared to the control serum (control) (*n* = 6).

**Figure 3.**
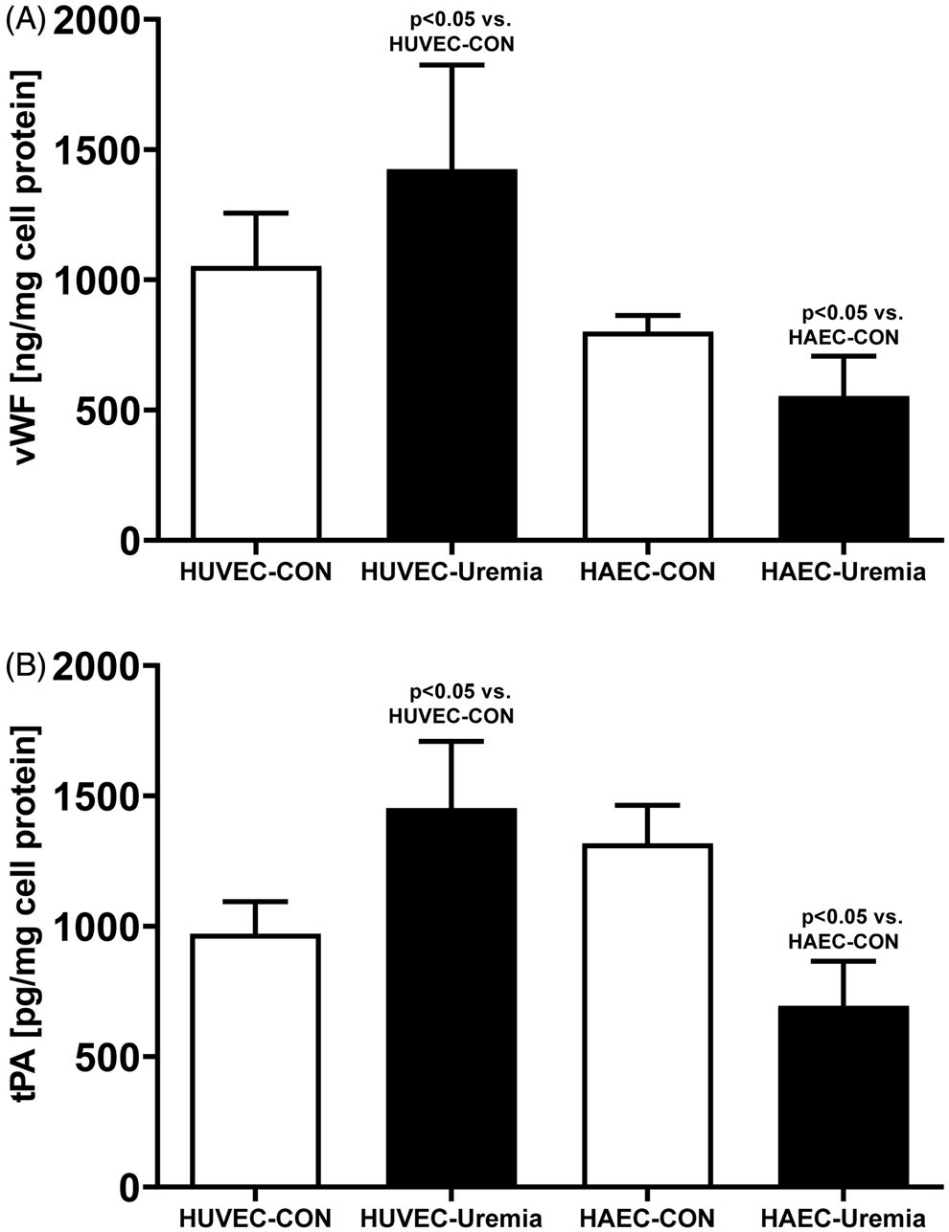
Synthesis of vWF (A) and t-PA (B) in human umbilical endothelial cells (HUVEC) and human aortic endothelial cells (HAEC) after exposure to uremic serum (uremia) as compared to the control serum (control) (*n* = 6).

Secretion of IL-6 was reduced in HUVEC by 38% (*p*<.05) and increased in HAEC by 55% (*p*<.05) (Figure 2(A)). Conversely, the synthesis of VEGF was increased in HUVEC by 64% (*p*<.001) and decreased in HAEC by 16% (*p*<.05) (Figure 2(B)). HUVEC exposed to uremic serum produced more vWF (+34%, *p*<.05) (Figure 3(A)) and t-PA (+53%, *p*<.05) (Figure 3(B)). The synthesis of these molecules was reduced in HAEC after exposure to the uremic serum by 31% (*p* < 05) and by 43% (*p*<.05), respectively (Figure 3(A,B)). Mean values of the secretory activity of the endothelial cells are presented in Table 2.

**Table 2.** Secretory activity of HUVEC and HAEC exposed to 20% uremic serum or 20% non-uremic serum.

	HUVEC-control	HUVEC-uremia	HAEC-control	HAEC-uremia
IL-6 (pg/mg cell protein)	169 ± 43	105 ± 17 *p*<.05 vs. HUVEC-control	279 ± 26	432 ± 57 *p*<.05 vs. HAEC-control
VEGF (pg/mg cells protein)	112 ± 24	184 ± 49 *p*<.001 vs. HUVEC-control	112 ± 25	94 ± 19 *p*<.05 vs. HAEC-control
vWF (ng/mg cell protein)	1013 ± 202	1414 ± 401 *p*<.05 vs. HUVEC-control	801 ± 63	553 ± 153 *p*<.05 vs. HAEC-control
t-PA (pg/mg cell protein	971 ± 123	1486 ± 256 *p*<.05 vs. HUVEC-control	1218 ± 118	695 ± 143 *p*<.05 vs. HAEC-control

## Discussion

The results of our study show significant differences in the effect of the uremic serum on the function of human venous and arterial endothelial cells. With the exception of IL-6 synthesis, the secretory activity of VEC was increased and the opposite effect was seen in AEC (Figure 1). That difference could be due to the stronger susceptibility of VEC, contrary to the endothelial cells, to oxidative stress [14]. During uremia various factors present in blood, such as uremic toxins, angiotensin II, endotoxins, byproducts of oxidative stress, can induce oxidative stress in endothelial cells [15]. The results from our study show that uremia has a minor effect on arterial endothelial cells but strongly modulates the functional properties of VEC. Previously, Dou et al. found that the uremic solute Indole-3 acetic acid caused the identical activation of COX-2 mRNA expression in venous, arterial, and microvascular endothelial cells [16]. However, these authors studied only one component of the uremic serum, and we exposed cells to all uremic toxins present in serum. Methe et al. described the differences in expression of the adhesive molecules between venous and arterial endothelial cells after their exposure to shear stress and inflammatory mediators [17]. In another study, venous and AEC treated with cobalt and titanium oxide nanoparticles produced not only different amounts of adhesive molecules, but also inflammatory cytokines [18].

In our experiments, in AEC only IL-6 synthesis was increased, and the consequence of that effect can be the acceleration of atherosclerosis [19]. Previous studies suggest that activation of the ubiquitin–proteasome pathway in AEC during their exposure to uremic serum plays an important role in the stimulation of their inflammatory response [20]. On the other hand, the suppression of PECAM-1 expression in AEC observed in our experiments may slow down the progression of atherosclerosis [21].

Our results show that VEC may play an important role in initiating various intravascular pathological processes in patients with end stage renal failure. We found changes in the gene expression as well as the secretory activity of these cells which are characteristic for the pathophysiology of chronic venous disease, such as increased expression of PECAM-1, and expression and secretion of VEGF [22] and vWF [23]. Previously, Serradell et al. described increased expression of adhesion molecules ELAM-1, VCAM-1, and ICAM-1, as well as increased presence of vWF in the extracellular matrix derived from VEC cultured in the presence of the uremic serum [24]. In uremic patients, these above-described changes in the properties of VEC, together with released microparticles loaded with TF from these cells [25], damage to the endothelial glycocalyx [26], and uremic thrombogenic toxins [27] may predispose patients to thromboembolic disorders. Mahmoodi et al. demonstrated that decrease of the GFR, even in non-uremic patients, increases the risk of venous thromboembolism [28]. In fact, the risk of spontaneous thrombosis in patients with stage 3 or 4 chronic renal failure is doubled as compared to the healthy control group [29]. Modification of VEC’ prothrombotic profile with adequate therapy may reduce that risk. In our recent study, we found that both N-acetylcysteine and sulodexide reduce the prothrombotic effect of uremic serum in VEC [30].

In summary, we found distinct reactions of venous and arterial endothelial cells to the uremic serum, with significant prothrombotic changes in the venous cells and inflammatory reaction in the aortic cells. During evaluation of the uremic effect on the function of endothelial cells, differences between the cells originating from the various parts of the vascular system should be taken into consideration.
